# Towards Less Plastic in Food Contact Materials: An In-Depth Overview of the Belgian Market

**DOI:** 10.3390/foods12142737

**Published:** 2023-07-18

**Authors:** Salvatore Ciano, Mélanie Di Mario, Séverine Goscinny, Els Van Hoeck

**Affiliations:** Scientific Direction “Chemical and Physical Health Risks”, Sciensano, Rue Juliette Wytsman 14, 1050 Ixelles, Belgium; melanie.dimario@sciensano.be (M.D.M.); severine.goscinny@sciensano.be (S.G.); els.vanhoeck@sciensano.be (E.V.H.)

**Keywords:** plastic alternatives, food contact materials, packaging, market study, consumer preferences, paper and board packaging, disposable plastic, bioplastic, silicones, recycled plastic

## Abstract

The food contact materials (FCMs) industry is forced to develop substitute materials due to constant pressure from consumers and authorities to reduce fossil-based plastic. Several alternatives are available on the market. However, market share, trends, and consumer preferences are still unclear. Therefore, this study aims to provide an overview of the Belgian FCMs market, the available substitute materials, and their uses. The market analysis was performed with an integrated web-based approach. Fifty-two sources were investigated, covering e-shops selling materials intended to replace disposable plastic materials or being advertised as environmentally friendly and websites describing homemade FCMs. The first screening identified 10,523 articles. The following data cleaning process resulted in a homogeneous dataset containing 2688 unique entries, systematically categorised into fifteen material categories and seven utilisation classes. Paper and board was the most popular material category (i.e., 37% of the entries), followed by bagasse, accounting for 9% of the entries. Takeaway and food serving (44.4% and 22.8% of the entries) were the most common usage categories. The study pursued to provide insights into current trends and consumer preferences, highlighting priorities for safety assessment and future policy making.

## 1. Introduction

Food is constantly in contact with many materials from farm to fork. These materials are called food contact materials (FCMs) and include transportation containers, machinery, kitchenware, and packaging [1]. Plastics are, by far, the most used material for FCMs because of their numerous properties like lightweight, malleability, resistance to corrosion, transparency, and water- or oil-proof characteristics. Moreover, plastics support the achievement of food safety, contribute to food waste reduction, and are cheap to produce [2]. For these reasons, plastic FCM applications have dramatically increased in the last decades and have become part of the modern lifestyle [3]. In 2021, the production of plastics was estimated at 57 million tons in Europe [4].

However, the production, use, and disposal of these materials can result in significant environmental damage and substantial contributions to carbon emissions. Kan and Miller revealed that plastic packaging accounts for up to 20% of greenhouse gas emissions associated with packaged food, with a landfill rate ranging from 61% to 77% in the U.S. [5]. Policymakers’ interest and awareness have grown worldwide, and different initiatives have been implemented to reduce the magnitude of the problem. In Europe, this focus is reflected in several actions. The European Green Deal, launched in December 2019, is a comprehensive plan to make the EU’s economy more sustainable. One of the key goals of the Green Deal is to move towards a circular economy, where resources are used more efficiently, and waste is minimised. Examples are the promotion of products designed to be durable and have an extended lifespan while also being repairable, recyclable, and reusable [6]. The Plastics Strategy, launched in January 2018, is another EU initiative aimed at reducing the impact of plastics on the environment. The strategy includes several measures to reduce the use of single-use plastics, including FCM. For example, the EU has adopted a ban on certain single-use plastic products, such as straws and cutlery, and has set targets for the use of recycled plastics in new products [2,7]. Lastly, The Circular Economy Action Plan, adopted in March 2020, includes key measures to promote sustainable FCM. These measures include using sustainable materials such as biobased, biodegradable, and recycled materials. Additionally, the plan encourages the design of FCMs that are easy to disassemble and recycle, as well as measures to promote refillable and reusable FCM [8].

The consumer is also forcing the packaging industry to change. As consumers become increasingly aware of the environmental impacts of packaging waste, they are demanding more sustainable and eco-friendly packaging options. In addition to activism, consumers can drive change through their purchasing decisions. By choosing products with more sustainable packaging, consumers can signal to manufacturers that there is a market for these products. This demand is placing pressure on manufacturers to develop and adopt more sustainable packaging practices, and one of the primary market responses has been the increasing availability of substitute materials for plastic in FCMs [9,10,11].

Therefore, FCMs made of natural origin materials and/or biodegradable polymers (also known as biopolymers) are rising in popularity. Biodegradable plastics are polymeric materials in which at least one step in the degradation process is through metabolisation with naturally occurring organisms. On the other hand, biopolymers can be broadly divided into different categories: (i) natural biopolymers such as plant carbohydrates and animal or plant origin proteins; (ii) synthetic biodegradable polymers; (iii) biopolymers produced by microbial fermentation like microbial polyesters [12]. Biopolymers are often combined with additives and functional nanostructures to overcome technical limitations so that they can be used for various FCM applications [13].

Due to their eco-friendly reputation, paper-based materials have been widely used in food packaging for products like milk, beverages, powders, confectionery, and bakery items. They are mainly used in primary packaging (in direct contact with the food) and secondary packaging (for transporting and storing primary packages). However, plain paper has limited functionality, such as poor barrier properties, low heat sealability, and insufficient strength for food products. Therefore, paper is often treated with additives or laminated with aluminium or plastic to enhance its functional properties [14].

In addition, new options are proposed in vegetable fibres, textiles, wood, or wooden-like materials (e.g., bamboo). They are biodegradable, renewable, and have good strength and barrier properties. However, they may require chemical treatments to improve their functionality (e.g., limited durability and resistance to moisture or heat), and the environmental impacts of the production of these materials are still under investigation [15].

Finally, the popularity of food used as FCMs is rising. It is also called edible FCMs, which refers to FCMs made from edible substances, such as starches, proteins, and other natural materials [16]. These materials are designed to be consumed along with the food they are in contact with, reducing waste and providing a more sustainable alternative to traditional FCMs [17]. However, their application is limited to specific food items and may not provide the desired barrier properties for all types of food [17].

Another growing trend is represented by homemade FCMs, with many websites that provide instructions for making domestic packaging or containers. Examples include cloth napkins, reusable beeswax wraps, or coffee filters.

Despite the availability of plastic alternatives for FCMs, a detailed market description is currently missing. Some reports from market agencies are available, focusing on the market share of packaging [18] or the global food packaging market [19,20]. However, to the best of our knowledge, this is the first study exploring the market of FCMs substitutes for plastic. First, an in-depth market survey mapped all recent trends (i.e., substitute materials) in the Belgian market. Next, the results were collected in a database, and the most commonly occurring materials and usage categories were identified. Finally, the future applications of this database were investigated.

## 2. Materials and Methods

### 2.1. Study Design

A typical market investigation process involves four main phases: (i) definition of the problematic and study scope(s); (ii) research plan development; (iii) collection and treatment of the data; (iv) interpretation and communication of the results [21]. This research aimed to evaluate the market of plastic alternatives for FCMs in Belgium. For this study’s purposes, FCMs plastic alternatives are defined as emerging FCMs (e.g., bagasse, palm leaves, etc.) or as classical FCMs (e.g., metals) used for “new” applications, intended to replace plastic items (i.e., metal pans were not considered in the study, while metal straws were). Moreover, the study did not include packaging already in contact with foods. The full investigation of all types of FCMs available on the Belgian market was not within the study’s scope.

The study was based on the analysis of the web market offer using specific keywords. Manual web data extraction can be resource- and time-consuming; however, web scraping tools provide an easy-to-use way to pile up and organise information [22,23].

The data were collected in a matrix, following a data cleaning step. Data cleaning is the process of identifying and correcting or removing errors, inconsistencies, and inaccuracies in a dataset. It is an essential step in preparing data for analysis, as the data quality can significantly affect the accuracy and reliability of the results. It can be manual or automated and typically involves several steps, including removing duplicates, handling missing values, fixing inconsistent values, removing outliers, and standardising data. [24,25].

The results were finally statistically treated and discussed.

### 2.2. Data Sources

Relevant retailers were identified online using the search engine Google (https://www.google.com), from a Belgian location (Brussels), in the period February–May 2022, using the following keywords (in French, Dutch, or English): *Sustainable, Biodegradable, Anti-microbial, Natural, Green, Zero-waste, Reusable, Environmentally friendly, Eco-friendly, Recycled,* and *Compostable.* Additional retailers were added based on expert knowledge. The results were filtered for retailers offering FCM, and only items not yet in contact with food were considered. The search was limited to websites able to ship in Belgium and homemade FCM websites in one of the official Belgian languages or English. The final list of 52 sources consulted for the study is given in Table S1.

### 2.3. Data Mining and Data Cleaning

The Web Scraper browser extension (https://www.webscraper.io/, accessed on 1–29 July 2022) was used to extract information (name, description, material(s), picture, and brand) from the websites, and the information was collected in a Microsoft Excel Open XML Spreadsheet (.xlsx file extension).

**Figure 1:**
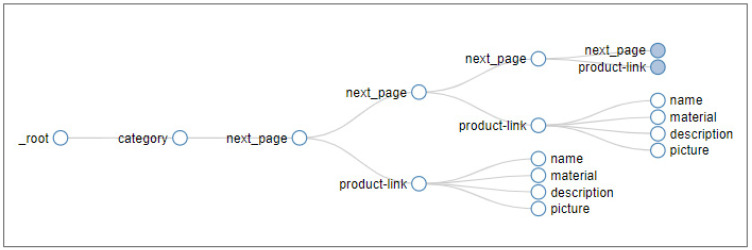
Biofutura website, sitemap selector graph, obtained with “Web Scraper” browser extension.

An example of a coded sitemap (for the website of the retailer Biofutura) is given in Supplementary Material (Code S1). Figure 1 shows the sitemap selector graph for the same website.

Next, a manual data cleaning step was needed to delete duplicates and nonrelevant items and allocate appropriate material and usage categories. This process involved several phases:

1. Removing duplicates: e.g., an identical product from different retailers, the same product in various sizes, and the same product in different versions (like knife, fork, or spoon).
2. Handling missing values, like the material description.
3. Deleting nonavailable items (assumed as nonavailable on the market).
4. Data standardisation: ensuring harmonised material and usage categories through the dataset.

The result was a matrix of 2688 entries containing the information displayed in Table 1.

## 3. Results and Discussion

The aim to minimise the environmental consequences of plastic had a disrupting effect on the FCM market. Market research was needed to identify alternative materials and their applications to understand consumer preferences and provide insights for policymakers, safety assessors, and researchers.

Market research is a set of techniques used in social sciences to collect, analyse, and interpret data concerning the conduct of market participants [27]. In this study, an in-depth market study was performed to identify all the new trends already available on the Belgian market intended to replace disposable plastic FCMs or advertised as environmentally friendly. Since online shopping is constantly growing, especially after the COVID-19 restriction [28,29], the market study focused on online shops, although some retailers also offer physical shops. Notably, only shops intended for direct consumers or small/medium businesses were consulted. Moreover, FCMs already in contact with food were excluded. The objective of the market study was not to obtain an all-encompassing census of all possible substitute FCMs available in Belgium but to provide a representative overview.

Once the research criteria were defined, 59 online shops were initially identified. However, a few websites were excluded due to unsuitability (nonpertinent shops, no online catalogue, lack of information). As a result, the final list contained 52 sources, with websites concerning homemade FCMs grouped as a single source. A total of 10,523 entries were collected in this phase.

When combining information from different sources, repetition or mislabelling of data can be expected. Data cleaning is the procedure of adjusting or eliminating improper, incomplete, or duplicate data within a dataset [24,25]. This process involved several steps in the current study, including removing duplicates and nonavailable items and assigning consistent material and usage categories. After the data cleaning process, the dataset comprised 2688 unique entries. However, several difficulties were encountered during the data cleaning. First, retailers have no harmonised vocabulary. It is the case for the “compostable” feature, which is not always specified under which conditions (industrial or home composting). Also, it was challenging to understand what kind of bioplastic the items were made of. Bioplastics can be biobased and biodegradable or only have one of these features [30]. Lack of harmonisation was present for paper and board, also. According to different retailers, the same material could be referred to as paper, kraft or pulp. However, these features were not always well described on the websites. Secondly, the offer seemed volatile. Around 70% of the products had no brand, and others, with the same claimed characteristics and applications, could easily replace them. Lastly, only limited information was declared, and crucial information was missing (e.g., brand, presence of a coating, the intended use, or origin).

Next, the entries were classified into 15 material categories. This step was critical to ensure consistency and accuracy in data analysis. By sorting the dataset this way, it was possible to compare and contrast different materials and identify patterns or trends in the data. The rationale beyond the classification followed Annex I of the European Framework Regulation (EU) No. 1935/2004 [31]. Since this Regulation is not specific enough, additional material types were added: paper analogues (e.g., bagasse and wheat pulp), stone and wood analogues (e.g., bamboo, palm leaves, coconut, reed, straw, wheat). The “food used as FCM” material category was also added, even if these items comply with the definition of food under the general food law [32].

Moreover, a distinction was made between recycled plastic and bioplastic. Both are plastic polymers undergoing the same FCM European Regulation [33]. Nevertheless, recycled plastic refers to plastic waste that has been processed and reconstituted into new products. It must also comply with an ad hoc European Regulation [34]. Instead, bioplastics are virgin polymers either obtained from biomass or biodegradable (or both) [35,36]. However, the lack of consistent definitions discussed earlier resulted in different labelling by the retailers. Examples were biodegradable bioplastic, compostable bioplastics, biobased polymers, etc. This study grouped all these materials as “bioplastic”. Finally, particular/niche materials (e.g., recycled sunflower and rapeseed oil, ocean-bound recycled plastic, seaweed, or rice hulls) were classified as “special cases”. These items were made of materials unable to be assigned in the 14 categories mentioned earlier or worthy of being discussed separately.

**Figure 2:**
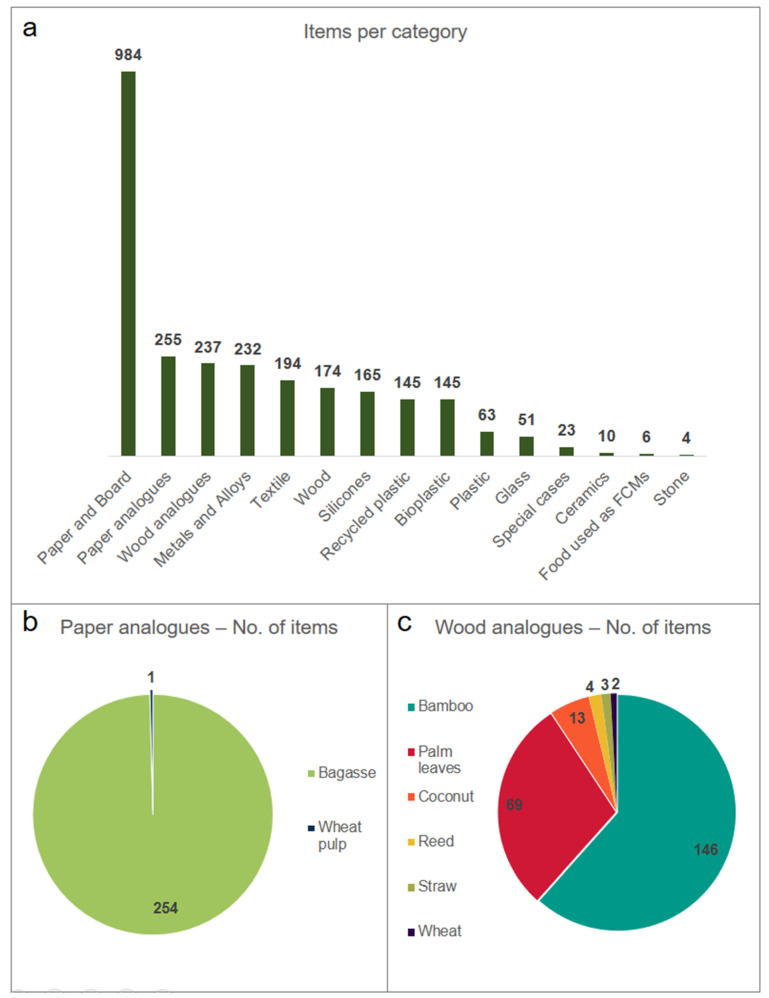
Overview of the materials used to manufacture alternative FCMs. (**a**) Number of samples per material. Bagasse and wheat pulp were grouped in the “paper analogues” category. Bamboo, palm leaves, coconut, reed, straw, and wheat were grouped in the “wood analogues” category. (**b**) Distribution of the paper analogues. (**c**) Distribution of the wood analogues.

An overview of the distribution of the materials is given in Figure 2.

Some interesting observations can be made. First, the material distribution was not homogeneous. The first seven most commonly occurring materials (paper and board, paper analogues, wood analogues, metals and alloys, textile, wood, and silicones) accounted for 83% of the occurrences. In comparison, eight materials shared the remaining 17%. The most commonly occurring substitute material was paper and board (36.6% of the total), which is comparable with the global market share for paper and paper-based food packaging (value share of nearly 32% in 2019) [20]. Moreover, the consumer preference for paper and paper analogues (paper, bagasse, and wheat pulp) can be pointed out since they accounted together for almost 50% of the resulting items, confirming the consumer perception of paper packaging as the most eco-friendly choice [37,38].

For 73.2% of the paper and board products, no information on coating (presence, absence, or material description) was provided to the consumers, who could not know which substance was actually in contact with their food. The materials used for coating paper vary, from plastic or bioplastic to wax-based materials. The coating provides a protective barrier against moisture, grease, and other substances. It also improves the packaging’s strength, making it more resistant to tearing or puncturing during transportation and handling [39]. However, some risks are associated with coated paper packaging since the coating could be a source of genotoxic contaminants [40].

A trend that can be pointed out is the rise of wood analogues material. Bamboo, palm leaves, coconut, reed, straw, and wheat possess different properties, allowing multiple applications (single- and repeated-use) and account (together) for 8.8% of the results. These items were made entirely from natural materials and should not be confused with melamine tableware, where bamboo is used as a natural filler, since their use has been banned in the EU because these fibres are not authorised additives, and safety issues were reported [41].

It is also interesting to note that recycled plastic and bioplastic represented only 5.4% of the results each. Specific policies and regulations have been set for recycled plastic packaging [34]; however, the market response seems tepid. The reason is unclear, but Testa et al. demonstrated that consumers still consider recyclable, recycled, or compostable plastic packaging as equivalent solutions, unaware of the different environmental consequences [42]. However, it should be noted that fossil-based plastic materials, previously marketed as single-use plastic, are still available on the market, but now these products are claimed as “reusable” and marketed as more sustainable. This type of labelling is misleading the consumer and should be avoided.

Silicone products accounted for 6.1% of the overall items, indicating an emerging trend. Silicone is a synthetic rubber-like material made from silicon, oxygen, carbon, and other elements. The use of silicone in tableware and cookware has become increasingly popular in recent years due to its many advantages, like heat resistance, nonstick properties, durability, and versatility. It is generally considered a safer alternative to other materials because, for example, it is free of bisphenol A. However, other substances could migrate to the food, and their safety is still under investigation [43].

Twenty-three products belonged to the category “special cases”. These items, with diverse applications, were made of innovative materials, like seaweed, or they were the results of niche applications of bioplastic (e.g., combination of PLA and starch, eucalyptus tree), recycled plastic (ocean bound), or combinations of plastic with other natural source materials (coffee husk, rice hulls).

Finally, six items belonged to the category of food used as FCMs, like straws, cutlery, and cups. Even if not covered by the Framework Regulation (EU) No 1935/2004 on FCMs, they represent an alternative to single-use plastic with increasing consumer acceptance [44].

**Figure 3:**
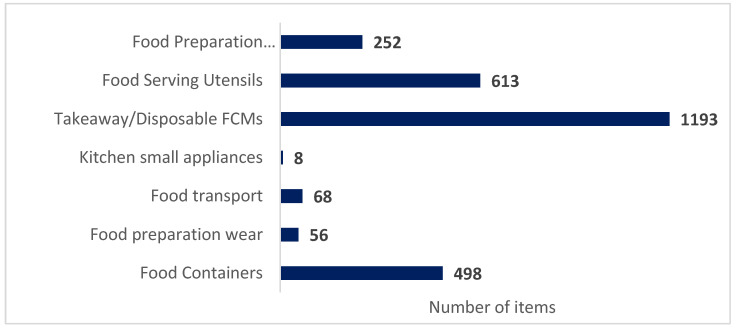
Results according to the item’s intended use.

Next, the dataset could also be classified according to the item’s intended use. This classification was based on the kitchenware guidelines of the European Reference Laboratory (EURL) [26]. The dataset was organised into the following categories: (i) food preparation wear, (ii) food preparation utensils/implements, (iii) food serving utensils, (iv) food containers, (v) kitchen small appliances, (vi) food transport, and (vii) takeaway/disposable FCMs. The last two categories are not mentioned in the kitchenware guideline but were added because of their relevance to the current investigation. The takeaway/disposable category was built with items that could fit in one of the categories mentioned above (for example, food serving, food container, or food transport) but with clear single-use and food-delivery vocations. The same principle was applied for those containers clearly intended for transport instead of storage and then categorised as food transport. Figure 3 gives an overview of the usage categorisation results.

The most represented category was “takeaway/disposable FCMs” (44.4% of the total entries). These materials are commonly used in fast-food chains, restaurants, and other food service industries requiring quick and convenient packaging. In recent years, the use of takeaway or disposable FCMs has been increasing due to the growing demand for convenience and portability [2], particularly during the COVID-19 pandemic [45]. However, there is also a growing concern about the environmental impact of these materials, as they generate a significant amount of waste that ends up in landfills and contributes to pollution. This issue led to the ban on single-use plastic in the EU mentioned above [7]. As a result, this class represents the most significant change in the FCM scene. In light of the pressing need to replace single-use plastics, considerable research is being conducted to explore alternative materials [46,47,48,49]. Among the most common alternatives in Europe are paper and other plant-based materials, including bamboo, palm leaves, and moulded plant fibres [50].

**Figure 4:**
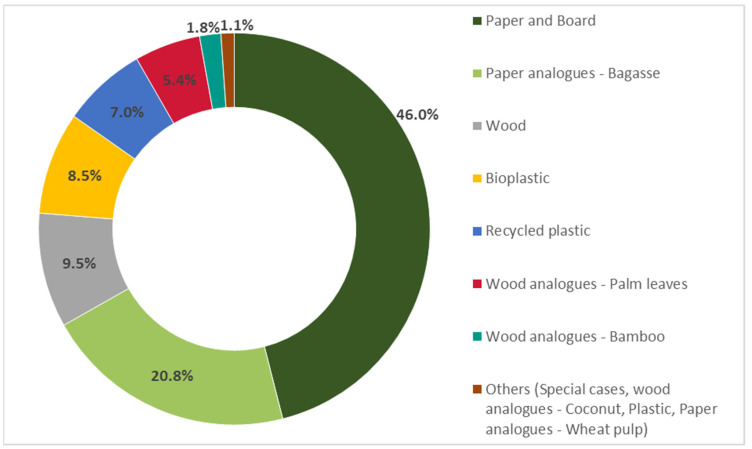
Takeaway/disposable FCM, percentage of material distribution.

An overview of the materials used for manufacturing takeaway/disposable FCMs is presented in Figure 4.

For this category, the most used alternative on the Belgian market was paper and board, which accounted for more than 46% of the class, followed by bagasse (20.8%), wood (9.5%), and bioplastic (8.5%). The takeaway/disposable category represented by far the primary application for these categories, with 58%, 98%, 65%, and 70% of the total occurrences of paper and board, bagasse, wood, and bioplastic, respectively. This material distribution can also be related to the specific properties of the materials. For single-use and takeaway applications, limited moisture and heat resistance is usually required, fitting the limited durability of these materials.

On the opposite side, the “kitchen small appliances” accounted for only 0.3% of the dataset, proposing the products of this category as less inclined to material swap. In addition, it can be stressed that one of every four food containers inventoried was made of textile (64% of the textile samples belong to this category). Finally, it is interesting to highlight that due to intrinsic characteristics, the food serving category showed the highest number of material types (all the different materials categories and subcategories, except wheat pulp).

## 4. Research Limitations and Perspectives

Although the study identified exciting trends regarding the material and application of FCMs, some limitations should be mentioned. First, the principal database limitation was the absence of information on brands/producers and materials. This could lead to market misrepresentation since the same item, marketed from different retailers, may appear as separate products. On the other hand, the absence of detail concerning the material used, especially for coatings, bioplastics, and recycled plastic, did not allow a higher level of detail. Secondly, there was a lack of information about sell volumes and research frequency. Sell volumes can help understand the actual market share of a certain material (even if the number of items is correlated with the market request), while repeating the same research over time would help trace exhaustive trends. Collaboration with FCM industries and sector associations would be critical for these aspects. It is also essential to recall that the study did not focus on the full Belgian FCM landscape but only on articles and materials placed on the market as such. Otherwise, packaging and FCMs already in contact with food were not included in the survey, prioritising end-users’ preferences rather than corporation choices. Finally, the current results can be integrated with market share data of substitute FCMs compared to plastic FCM, providing further interesting observations.

However, this market study fills a gap in describing the new FCM, or new applications of existing FCM, intended to replace articles made in plastic. The study can help businesses in several ways, such as identifying market opportunities, evaluating the competitive landscape, assessing market growth potential, identifying key drivers and challenges, and developing product and marketing strategies. In addition to the business-related aspects, insights from this research can help guide policy decisions related to waste reduction, recycling, and sustainability and help safety assessors set priorities based on market demand and potential exposure levels. Labels for substitute materials often display “sustainability claims” (e.g., “natural”, “biodegradable”, “sustainable”, “eco-friendly”, etc.) but a comprehensive evaluation of their environmental impact throughout their lifecycle is still missing and is challenging to realise [51,52,53]. Furthermore, the health risks associated with these substitute materials are largely unknown and related to contaminants and allergens that may be present, with no solid rules addressing specific contaminant release set at the European level, unlike for plastic, which is covered by Regulation (EU) No. 10/2011 [54,55].

Finally, the study can help researchers to identify gaps in the current knowledge of alternative FCMs to plastic and prioritise future research efforts.

## 5. Conclusions

The FCM market has transformed because of several key external drivers. The present study aimed to answer the following research question: What are the new trends to replace fossil-based plastic FCMs or being presented as environmentally friendly on the Belgian market? An in-depth market study was performed, and a database of substitute materials products was constructed. A critical manual finishing of the results was necessary to tackle the challenges like the absence of coordinated vocabulary between retailers, lack of information on the website (e.g., brand, coating, etc.), and high market volatility. Eventually, the database contained 2688 unique entries from 52 sources. Paper and paper analogues dominated the market of alternative materials, with the surprising presence of plastic, now more sustainable. Recycled plastic and bioplastic did not show any promising trend, probably due to the confusing and nonharmonised terminology that does not help to communicate their sustainability. Wood analogues (bamboo, but also coconut, reed, and palm leaves) can be identified as a new trend and deserve more profound investigation. The takeaway/disposable FCMs were more prone to alternative materials, with kitchen small appliances on the opposite side. The results aspire to help the discussion of stakeholders and policymakers for improving regulations and the safety assessment of these materials.

## Figures and Tables

**Table 1:** Information collected in the matrix.

Info	Description
ID code	A unique identifier code.
Material	Information on the material as described in the data source.
Main material classification	According to a harmonised material classification.
Material subcategory	If needed (e.g., bagasse, bamboo, coconut, etc.).
Coating	Presence of a coating, only for paper and board, and according to the information described in the data source.
Secondary material	In case different materials are used/declared.
Additional information	
Main usage category	Based on EURL kitchenware guidelines [26].
Usage subcategory	Based on EURL kitchenware guidelines [26].
Usage detail	If needed (e.g., disposable, etc.).
Description	Description of the FCM as available in the data source.
Brand	Brand of the item (according to data source).
Retailer	Name of the shop.
Source	Link to the website.
Remarks	

## Data Availability

The data presented in this study are available on request from the corresponding author. The data are not publicly available due to privacy restrictions.

## Supplementary Materials

The following supporting information can be downloaded at: https://www.mdpi.com/article/10.3390/foods12142737/s1, Table S1: Final list of websites included in the market study; Code S1: Example of a coded sitemap.
