# Supplementary material for: Towards Less Plastic in Food Contact Materials: An In-Depth Overview of the Belgian Market
Source: Foods. 2023 Jul 18;12(14):2737. doi: 10.3390/foods12142737 (PMC10379060; doi:10.3390/foods12142737)
Supplement: Supplementary file 1 [file foods-12-02737-s001.zip › foods-2515747-supplementary.pdf]

Table S1

Final list of websites included in the market study
NB: the home-made websites have been considered as a unique source

Retailer	Physical shop available	url
a Healthy Life		https://www.ahealthylife.nl
ACE Packaging & Technics		https://www.acepackaging.be
AVA	x	https://www.ava.be
Beeswrap		https://www.beeswrap.com
Bewust Puur!		https://www.bewustpuur.nl
Big Green Smile		https://www.biggreensmile.nl
Bio Degradable Packaging BVBA		https://biodp.eu
Bioodi		https://www.bioodi.fr
Biodisposable		https://biodisposables.shop
Biofutura		https://www.biofutura.com
Biopack		https://www.biopack.be
Blabloom	x	https://www.blabloom.com
bol.com		https://www.bol.com
Boobalou		https://www.boobalou.co.uk
CASA	x	https://be.casashops.com
Cookserve	x	https://www.cookserve.be
Dagelijks duurzaam		https://dagelijksduurzaam.be
Decathlon	x	https://www.decathlon.be
Denico		https://www.denico.eu
Dille & Kamille	x	https://www.dille-kamille.be
Dreambaby	x	https://www.dreambaby.be
EcoCompany		https://eco-company.nl
Ecolinq		https://www.ecolinq.com
Eco-logisch	x	https://www.eco-logisch.nl
Ecomondo		https://www.ecomondo.nl
Ecotheek		https://www.ecotheek.be
Garden4less	x	https://www.garden4less.co.uk
Gifi	x	https://livraison.gifi.fr
Grandado		https://www.grandado.com
Greenjump		https://www.greenjump.nl
Home-made		https://www.attainable-sustainable.net; https://thefarmchicks.typepad.com; https://www.diynatural.com;

		http://wellnessmama.com; https://aromalifestyle.nl; https://chevronstitches.blogspot.com; https://mamawordtgroen.blogspot.com https://zerowastechef.com
Jourdefete	x	https://www.boutique-jourdefete.com
Kleine Zebra		https://www.kleinezebra.com
Kudzu		https://www.kudzu.be
La foirfouille	x	https://www.lafoirfouille.fr
Lebonembalage		https://lebonemballage.com
Leven Zonder Afval		https://levenzonderafval.com
Makro	x	https://www.makroshop.be
Moonen Direct		https://www.moonendirect.nl
Nisbets		https://www.nisbets.be
Notpla		https://notpla.shop
Oil&Vinegar	x	https://www.oilvinegar.be
Oxfam	x	https://shop.oxfamwereldwinkels.be
Raja		https://www.rajapack.be
Retif	x	https://fr.retif.be
Sebio		https://sebio.be
Strawz		https://nl.strawz.eu
t Ecoloogje	x	https://www.ecoloogje.be
Thouy		https://www.thouy.net
Variapack		https://www.variapack.be
Versupack		https://www.versupackstore.com
Vuur en rook	x	https://www.vuurenrook.nl

Code S1

Example of a coded sitemap (for the website of the retailer Biofutura) with the Web Scraper browser extension (https://www.webscraper.io/) :

{"_id":"biofutura","startUrl":["https://www.biofutura.com/fr/votre-
activite"],"selectors":[{"id":"category","parentSelectors":["_root"],"type":"Selector
Link","selector":"[data-label='Votre activité'] .level-1 > li >
a","multiple":true},{"id":"next_page","parentSelectors":["category","next_page"],"
paginationType":"auto","selector":"div.toolbar:nth-of-type(2)
a.action","type":"SelectorPagination"},{"id":"product-
link","parentSelectors":["next_page"],"type":"SelectorLink","selector":"a.product-
item-link","multiple":true},{"id":"name","parentSelectors":["product-
link"],"type":"SelectorText","selector":".product-info-main
span[itemprop='name']","multiple":false,"regex":""},{"id":"material","parentSelect
ors":["product-link"],"type":"SelectorText","selector":".material-desc
div","multiple":false,"regex":""},{"id":"description","parentSelectors":["product-
link"],"type":"SelectorText","selector":".value
p","multiple":false,"regex":""},{"id":"picture","parentSelectors":["product-
link"],"type":"SelectorImage","selector":".slick-current img.gallery-
image","multiple":false}]}
